# Learning Active Implementation Frameworks: the role of implementation teams in a case study from Pakistan

**DOI:** 10.1080/16549716.2020.1805164

**Published:** 2020-08-25

**Authors:** Saima Hamid, Sheh Mureed, Aasia Kayani, Kiran Javed, Adnan Khan, Sayema Awais, Neelam Khan, Fakiha Tus-Salam, Dean L. Fixsen

**Affiliations:** a Fatima Jinnah Women University, Faculty of Social Sciences, Rawalpindi, Pakistan; b Maternal and Child Health Department, Health Services Academy, Islamabad, Pakistan; c Obstetrics and Gyna Ecology Department Holy Family Hospital, Rawalpindi, Pakistan; d Active Implementation Research Network, Chapel Hill, North Carolina, United States

**Keywords:** Implementation science and practice, capacity building, implementation team, fidelity, WHO ‘s safe childbirth checklist, Pakistan

## Abstract

In Pakistan, although coverage of Maternal, Newborn, and Child Health (MNCH) services has increased, the attributable disease burden remains high, indicating quality of these services remains suboptimal. To address this quality gap, challenges associated with the implementation of MNCH services will need to be addressed and effective use of the various MNCH guidelines will need to be supported, evaluated, and continuously improved. Even though the application of the field of implementation science and practice in the low- and middle-income settings has been limited, it is our belief, based on the experience described in this article that these competencies could enhance health professionals’ ability to, not only successfully integrate MNCH guidelines into health systems, but to also support their effective and sustainable use. To address this capacity gap in Pakistan, the Health Services Academy, as a member of the World Health Organization’s Human Reproduction Program (HRP) Alliance for Research Capacity Strengthening (RCS), has engaged, over the course of 16 months, in the ‘Implementation for the Professional Learner Program’ in 2019. This innovative implementation science and practice capacity-building program is developed and conducted by The World Health Organization (WHO) Collaborating Centre for Research Evidence for Sexual and Reproductive Health at the University of North Carolina at Chapel Hill (UNC). The initial cohort of this Program also included Palestine’s West Bank, and Egypt. The objectives of this Program were to cultivate implementation science and practice competencies, and to support the development of national, community-based or institution-based implementation teams. The expected outcomes of this program included, further enhancement of the capacity of local health professionals in implementation science, systemic change and the effective use of innovations in practice at sub-national/regional levels.

## Background

Despite having made unprecedented improvement in health service coverage in the past few decades, Pakistan is still lagging behind in the achievement of the milestones set for Maternal, Neonatal and Child health (MNCH) [1]. According to the Pakistan Demographic and Health Survey (PDHS), skilled birth attendance has leap frogged from a mere 38.8% in 2006–7 to a high of 69.3% in 2017–18 [2]. Nonetheless, the health indicators have been stubbornly unchanging, with neonatal mortality rate dropping only marginally from 54 per 1000 live births in 2006–7 to 42 per 1000 live births in 2017–18 [2]. The World Health Organization is cognizant of this mismatch between the increase in health service coverage and reduction in the burden of disease citing poor quality of care (QoC) as the major underlying determinant. As shown by the findings of the WHO Multicounty Survey on Maternal and Newborn Health, a wider health service coverage must be matched by concomitant improvements in quality of care if maternal mortality is to be reduced substantially [3]. Use of implementation science methods, including the Active Implementation Frameworks [4] and quality improvement iterative adaptation are examples of how evidence-based implementation strategies can be adapted, applied, and sustained within programs to strengthen maternal health services, hence improving QoC [4].

The purpose of implementation science and practice is to ensure the full and effective use of an innovation in practice in order to achieve socially significant outcomes [4]. The emerging field of implementation [5], has the potential to address the multiple issues leading to poor quality of maternal health services [6]. However, in low- and middle-income countries there remains limited understating what implementation is and the role it can play in maximizing the benefit of the interventions in use [7]. Studies of the competencies associated with this field have been emerging based on its extensive application in multiple contexts, including education and health in the high-income countries [8,9]. Although the application of the field of implementation science and practice in the low- and middle-income settings has been limited [7], it is our belief, based on the experience described in this article that these competencies could further develop health professionals’ ability to, not only successfully integrate MNCH guidelines into health systems, but to also support their effective and sustainable use.

Currently, the recommended public health curriculum of Pakistan is largely missing implementation science and practice concepts [9]. Health Services Academy (HSA) is Pakistan’s premier national-level public health institute with a mandate to build public health capacity and generate evidence for public health policy and practice. Graduates from HSA are working in key positions at national and provincial levels within government and non-government organizations. Being one of the oldest national-level training institute, HSA is uniquely positioned to influence public health training, as well as public health practice. However, even within HSA there is a need to develop the implementation practice and science capacity. To address this gap, the MNCH Department of HSA enrolled in the *Implementation for the Professional Learner*–a multi-country implementation practice and science capacity-building program developed and conducted by the WHO Collaborating Centre at University of North Carolina (UNC), United States.

The aim of this paper is to share the learning and outcomes resulting from this experience.

## Commencement of this experience

Implementation for the Professional Learner is a program that has been developed and conducted by the WHO Collaborating Centre at UNC to advance professionals’ understanding and skills in implementation practice and science. The participation in the WHO’s Human Reproduction Program Alliance meeting at Accra in Ghana in November of 2017, and the following correspondence led to the engagement of the Health Services Academy in Islamabad, Pakistan, the Ain Shams University in Cairo, Egypt, and the Birzeit University in the West Bank, Palestine as the first three country institutions in this capacity development project. The goal of this program was to cultivate implementation practice and science competencies, and to form the first implementation team in each of the three participating institutions.

While the Collaborating Centre at UNC used its own core funding to conduct the training, coaching and webinars, and to provide resources, including a learning platform to support the learning process, the country institutions were advised to: (1) select members of their respective institutions to form the core ‘Implementation Team’; (2) initiate a project, so the team can apply their learning about implementation in real-time; and (3) engage in a nine-module course spanning a period of 52 weeks.

The lead of the project selected individuals based on the criteria of highly functioning teams- team players, committed, diligent, and having diverse backgrounds. The final team selected consisted of eight team members from MNCH department, HSA alumni and clinicians. The implementation team was composed of professionals of diverse background and experience, including public health professionals, clinicians, sociologists and economists

Under the guidance and advice of the Collaborating Centre at UNC, implementation of the ‘Safe Childbirth Checklist’ was scoped as the project to work on, and a project site was selected. The ‘Safe Childbirth Checklist’ (SCC) is an evidence-based tool for the improvement of quality services at the time of birth [10]. Having been previously endorsed in Pakistan, it is a simple checklist for providers to ensure same standard of services being offered at four points/pauses for women in labour – mainly at the time of admission, just before pushing, soon after birth, and before discharge.

The project site was not too far from the Health Services Academy, Islamabad at a government tertiary care hospital. Reducing the workload, cost and time were among the criteria used to ensure the project was feasible. The Head of Department of Gynaecology & Obstetrics supported the project and selected two members of her own faculty to become part of theIimplementation Team. This support has not only enriched the learning experience, but also added to the rigor and thoroughness needed to ensure the effectiveness and feasibility of the implementation strategies designed at the hospital. Moreover, the clinicians’ ‘insider perspective’ was crucial for the implementation of the project.

## Online course process: ‘implementation for the professional learners’

The Collaborating Centre at UNC has designed the Program to be highly practical – with participants applying lessons, as they go, to real-life situations and local projects. Professor Dean Fixsen was the lead facilitator for the first cohort, and the learning and coaching were delivered through a cloud-based Learning Management System (LMS) which provided a virtual classroom with the facility to access reading materials, videos, presentations and recordings of the teaching sessions uploaded as the course progressed. This user-friendly LMS displayed the tentative calendar of activities and also allowed the participants to post queries between the classes. At the beginning of each module, the participants were required to answer a short quiz which assessed their pre-existing knowledge, a short video and relevant readings which introduced the module. At the end of each module, a webinar was set up in which the key concepts were briefly explained. Discussion between the participants was encouraged and posted queries were duly addressed. Further to that, a ‘Coaching Call’ with each team was held independently that was more focused on the individual projects being pursued by each team.

The learning itself consisted of nine modules divided into the following sections: introduction (module 1), implementation practice (Modules 2–7) and implementation research (modules 8 & 9) followed by the last session in which each of the three teams presented their own project and how the learning had supported putting into practice the innovations/interventions that were centre to the projects.

The modules were well-designed and took the students through progressively more advanced concepts based on the Active Implementation Frameworks (AIFs) outcomes [4] teaching theory and practice in tandem. The assignments were considered doable by the participants despite most being engaged in their respective jobs and any problems that arose were promptly removed by active troubleshooting by the UNC team.

Short-term outcomes achieved were completion of the coursework; developing and functioning as an implementation team; and achieving the implementation project goals. Achievement of these outcomes was facilitated by the personal drive and commitment of the team members, and the dynamic leadership provided by the Pakistan team lead. The long-term goal of this initiative is to use this learning to develop others’ capacity in implementation practice and science.

### Developing the implementation team

One of the effective strategies we used to bond together as a team and to overcome our differences, was to gather together at the same place for coaching calls, and to attend weekly meetings to discuss the literature and promote usage of common implementation language, fine-tuning details of the project with relevance to the concepts learned during the coaching calls. As a result, the team’s roles and responsibilities evolved with the members ‘gelling together’, each one respecting and realizing the expertise of others. The once-a-month coaching calls, scheduled after working hours and graciously hosted at the residence of the team lead, were the highlight of the month. Not only were these instructive but also motivating with the guidance and encouragement needed to maintain the team’s morale, productivity and functionality. Each coaching call provided the team a chance to review the project, devise new strategies or modify old ones, trouble-shoot obstacles and review progress.

### Project outcomes

The objective of our implementation project was to implement the WHO’s SCC at Obstetrics & Gynaecology department of the hospital and routinize the effective use of the SCC in the department. The AIFs [4] learned in the modules provided the structure and methodologies needed for doing just that. These six Frameworks – Usable innovation; Implementation Teams; Implementation Drivers; Implementation Stages; Improvement Cycles; And Systemic Change function in a highly integrated manner and drive the implementation process through stages.

There are four stages of implementation: ‘exploration’ (assessing and creating readiness), ‘installation’ (amassing human and financial resources); and ‘initial implementation’ activities and outcomes (supporting the use of the innovation in practice) that eventually lead to ‘full implementation’ within organizations and systems (at least 50% of the practitioners in an organization meeting fidelity standards for using the innovation in practice) [4]. Implementation Stages as experienced and understood by the team were interactive, non-linear, and additive.

The project outcomes are described and discussed as the Implementation Team moved through the different stages of Implementation.

#### Exploration stage

In the exploration stage, the Implementation Team defined SCC according to the criteria of ‘Usable Innovation’ and developed a measure for assessing fidelity for checklist [4]. Useable innovation is defined according to 4 criteria (these are: a clear description of the innovation; identifying the essential functions; operational definitions of the essential functions; and a practical assessment of fidelity. The team were able to define SCC according to these criteria, after conducting an intensive literature review, and brainstorming sessions during the weekly meetings. As the new intervention, SCC was clearly described, its essential functions were described, operational definitions of essential functions were given and a fidelity score for SCC was devised (Box 1). The fidelity of SCC was complete filling of the form by each practitioner for each section over total number of forms inserted into the patients file on a given day.

Concurrently, the Implementation Team created readiness for SCC implementation at the departmental level. The need for SCC as a quality improvement tool was endorsed, and the tool was contextualized. The final version of the tool was approved by the senior faculty of the department. Moreover, the concept of fidelity and how it will be measured was introduced. Leadership to support this project was identified during the Exploration Stage.

#### Installation stage

The Installation Stage involves acquiring or developing the resources needed to fully and effectively engage in the new ways of work [4]. During this stage, the forms were printed and delivered to the hospital to be replenished on monthly basis by funds contributed by the Implementation Team members. A mechanism was established for periodic collection of the data, data entry, and data analysis. For feedback and guidance, data were regularly discussed during the coaching calls. Furthermore, during this stage, roles and responsibilities of team members were assigned (Who will do what; how will they learn to do it; how will they improve). The team also learned to use the Implementation Drivers in relevance to the project activities.

#### Initial implementation stage

**Figure 1.**
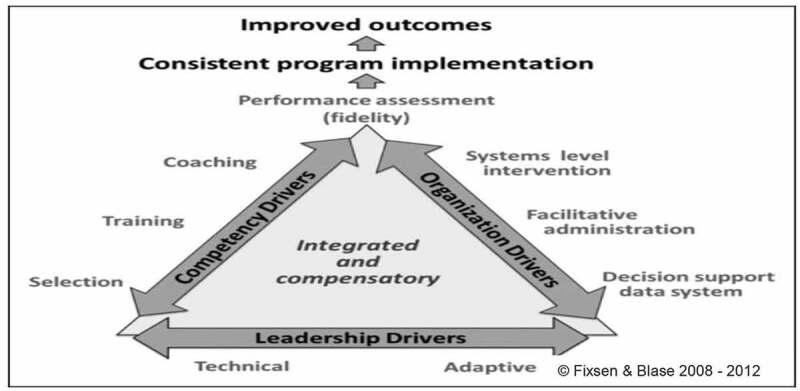
Implementation drivers [4,11].

The Initial Implementation Stage begins when the first practitioners start using the innovation for the first time [4]. The first SCC form was filled in March 2019. Effective use of Implementation Drivers is essential at this stage (Figure 1; reproduced from open access source) [4,11]. These drivers need to be in place and fully functioning to produce and sustain consistent uses of innovations by practitioners and reliable outcomes for the intended recipients of an innovation [4]. Competency of practitioners is ensured by appropriate selection, training, and coaching of the practitioners. In this project, the Implementation Team could not select the practitioners because of the organizational policies and structure. However, training of practitioners regarding implementation, content and filling of SCC forms and fidelity measures were conducted by Implementation Team members. Coaching was done to provide motivation and support to practitioners by Senior Registrars (SRs) in the process identifying barriers faced by the practitioners.

Organizational Drivers constitute one leg of Implementation Drivers framework. Facilitative Administration, Decision Support Data Systems (DSDS), and System Intervention are the three constructs of this driver [4]. By applying this driver, Implementation Team made communication links to share information with leadership and received their feedback. This DSDS model created by the team contained information on SCC fidelity, results of observations, and interactions with practitioners.

For Active Implementation, leadership must be identified, nurtured, and developed so that optimal outcomes are achieved and sustained [4]. Leadership Driver is used for addressing technical and adaptive challenges that arises during implementation. The Implementation Team role was to support leaders to identify technical challenges based on information from DSDS. And, to introduce changes in policy to assist practice (practice-to-policy loop). The adaptive challenges faced by our team require that a systemic change be brought about to lessen the burden on the practitioners to implement innovations in a more comprehensive way ensuring quality of care during the process of birth. This requires administrative changes like printing of new patient files with SCC principles of implementation incorporated within and increasing the number of practitioners, since managing several women in labour at the same time makes SCC filling an arduous job. However, these changes can be realized by engaging the higher echelons of power. The team is still trying to improve the facility conditions by engaging the institutional head to address these issues.

#### Full implementation

The Full Implementation Stage is reached when at least 50% of the practitioners in an organization meet fidelity criteria on a given day [4]. In other words, fully filled SCC forms (fidelity criteria), by 50% of the practitioners working in hospital on a given day. It often takes 2 to 4 years to reach Full Implementation when it is achieved at all [4]. As this project was started in March 2019, it would have been a remarkable feat had the implementation team met its target of full implementation within a period of one year (Table 1). According to Table 1, fidelity score for SCC filling was assessed in March and onwards. In the month of June, fidelity score was highest for 3 sections except for section 4 (before discharge). To increase fidelity scores Quality Improvement techniques were applied. In July 2019, fidelity score is seen dropping, the main reason being the change in leadership at the departmental level. Hence, the change put the project at a lesser priority level in the department. Nonetheless, multiple efforts were made to reach project objectives. Improvement cycles are discussed here as part of this stage because they were crucial to this project outcomes.

**Table 1.** Percentage of fully filled SCC forms for all sections by practitioners in the hospital from March to July 2019.

Month (n = forms inserted)/ Section	Fidelity Section I – On Admission %	Fidelity Section II – Just Before Pushing %	Fidelity Section III – Soon After Birth %	Fidelity Section IV – Before Discharge %
March(n = 64)	9	2	0	67
April(n = 181)	23	15	1	48
May(n = 494)	39	47	22	9
June(n = 542)	86	80	62	38
July (n = 366)	68	6	41	33

Fidelity score = Complete filling of the form by each practitioner for each section over total number of forms inserted into the patients file on a given day.

Improvement cycles are critical to continued use of effective innovations. They are based on trial and error learning approaches for problem solving in all aspects of implementation [4]. For this project PDSAC (Plan-Do-Study-Act Cycle) logic was used to improve the innovations (SCC), the implementation methods and the enabling contexts. The information from DSDS was used to determine progress and identify issues related to fidelity. When an issue was identified, a plan was developed, the plan was tested (Do), there data were reviewed to assess the impact of the plan (Study). Finally, a decision was made by the team to either continue with the plan/change or to restart the cycle (Act). After each iteration of PDSA cycles, the finding was shared with the leadership. As, when and where required leadership became integral part of the plan. A total of 6 PDSA cycles iterations were completed for this project (Table 2).

**Table 2.** Plan Do Study Act (PDSA) cycles for quality improvement for SCC implementation.

Iteration:	1^st^	2^nd^	3^rd^
**Dates:**	12 March 2019	24 April 2019	6 May 2019
**Issue Identified:**	Availability of SCC	Incomplete filling of SCC forms	Timely analysis and reporting
**Plan**	Make SCC form available. Increase understanding of SCC forms. To Improve coordination between HSA and hospital team members.	Scheduled meeting with the department and leadership. Meeting agenda, to present progress made so far to the leadership i.e. Head of Department (HoD). To, reconfirm commitments made by the leadership. Discuss challenges and solutions.	Increase coordination between the hospital team and HSA for collection of SCC forms and expediting data entry and analysis. Timely sharing of findings on WhatsApp group with leadership to identify and address barriers and challenges.
**Do**	Doctors were informed that SCC form were inserted into patient files by the nurses; WhatsApp group to increase communication within the team; Involvement of leadership during orientation and training sessions	Data on SCC filling was presented. HoD took notice of poorly filled forms. Group discussed time as a barrier in filling the form. Group reviewed the form and agreed that it did not require a lot of their time for filling.	Data forms to be collected through personal drivers on Mondays and Thursdays. Forms to be entered on the same day through Students Affairs staff of HSA. Analysis by HSA’s implementation team members latest by Friday morning. After endorsement by implementation team lead, sharing of data on WhatsApp group for onward transmission to HoD.
**Study**	Data entry and analysis using SPSS v.16; Review of data entry format in the weekly meetings	Review of fidelity data in the weekly meetings. Indicator shared and accepted by leadership: Frequency of SCC forms filled by each section over total number of forms entered in one-month time.	Internal review of the progress made on action points after one-month time. Time series plot of the fidelity measure using daily data. Marginal improvement in fidelity data.
**Act**	Data entry, analysis and feedback mechanisms established. WhatsApp group created for internal communication.	Consensus on diligently filling SCC was reached. HoD asked senior doctors to be proactive and motivate and support junior doctors. WhatsApp group to share data with leadership.	HoD recognized the need for further reinforcement and motivation of practitioners. SCC forms to be discussed in departmental morning meetings.
**Iteration:**	4^th^	5^th^	6th
**Dates:**	26 May 2019	26 July 2019	6 January 2020
**Issue Identified:**	Low fidelity scores and accessibility to SCC forms	Low fidelity scores	Change in leadership
**Plan**	To increase fidelity of the forms, Senior Residents (SR) at the department were assigned to support other practitioners in filling of the forms by providing advice and clarifications where needed. To increase visibility and accessibility of the SCC forms.	To identify barriers or challenges faced by practitioners in filling the forms. Qualitative data collection done by using participant observation in filling checklist.	To convince the new HoD to accept the new Patient file with incorporation of the SCC forms and to help facilitate the systemic change required.
**Do**	SR supported the practitioners in filling of the SCC forms for one week. The accessibility and visibility of the SCC forms were made easily available to the practitioners by placing them on racks next to the patient’s files.	A checklist was developed to assess practitioners if they were filling the form accurately.	A meeting was arranged with new HoD and the faculty of hospital to present the new Patient file.
**Study**	Review of fidelity data in the weekly meetings. Participant observation by hospital team to see if the SCC forms were easily accessible or not.	Interviews with practitioners to assess barriers. Checklist used to assess practitioner performance. Practitioners reported the extreme workload and lack of time for SCC form filling. Observers found that all the steps in the SCC were being performed but not documented in the checklists.	The new HoD accepted the new Patient file and said that this process needs to be formalized through proper channel.
**Act**	Involving SRs was beneficial in increasing fidelity score. However, this approach was not very sustainable due to busy schedule of SRs. Making the forms easily available increased number of forms being inserted into patients’ files. Yet, it didn’t have much impact on filling of the forms. It was decided to go for structured observations to explore the reason behind low fidelity scores.	The SCC form was proposed to be incorporated into the patient file to optimize access and easy filling of SCC forms.	The new HoD recommended that the institutional Head should be taken on board for the systemic change required; hence the institutional head needs to be approached and included in further proceedings to complete the practice-policy communication.

## Discussion

This case study illustrates the trials and tribulations faced by Implementation Team in the introduction of a new albeit evidence-based intervention and how a team newly instructed in the principles of implementation overcame the obstacles by using Improvement Cycles. The implementation barriers faced by our team have also been documented in literature including poor motivation of staff and resistance to change the status quo [12], in addition to poor or inaccessible checklist supply.

The uniqueness of this project under the ‘Implementation for the Professional Learner’ Program, lies in the fact that whatever was taught during the course was actually put into practice by the course participants. The identification and characterization of the ‘Usable Innovation’ followed by its contextualization and development of the fidelity scores led the participants to think in practical terms of its benefits and relevance. In order to achieve high fidelity, implementation drivers of competency, leadership and organization were used to assess their own performance and to identify areas demanding improvement. The crucial role of Implementation Team in the whole process was recognized and appreciated. Overcoming identified barriers by data-informed decision making through the use of PDSA cycles led to some improvement in fidelity scores. The team also learnt the vital role of systemic change (i.e. practice-to-policy feedback loop) in the sustainability of the innovation in the institution with a clear understanding that such change takes time.

This capacity-building initiative was undertaken by a few committed individuals who went over and above their routine work. The WHO Collaborating Centre at UNC’s implementation science capacity development program has challenged the teams’ mind sets and motivated them to identify ways to use implementation science and practice. Through online training Implementation Team acquired knowledge on AIFs. The continued coaching on SCC project provided the team an opportunity to translate knowledge into practice. Successful outcome of the project is reflected in SCC being embedded within routine patient records. However, Systemic Change proved to be very challenging due to change in senior leadership.

This project provides many practical lessons and transferable learnings for other public health professionals and implementation scientists. The coaching methodology adopted by the UNC team provides a good structure for replication at minimal cost by others. This approach enhanced indigenous capacity for implementation of public health professionals by utilizing digital platforms. This may be seen as an opportunity in the current COVID-19 pandemic times when acceptability towards virtual collaboration has increased. Moreover, the training underscored inculcating implementation science competencies, and developing local and regional Implementation Teams in programs and projects for expedited achievement of outcomes. Ensuring Implementation Teams comprise members from diverse backgrounds contributes greatly towards enriching perspectives on providing solutions to the challenges that are inevitably faced when implementing an innovation. Engaging leadership right from the exploration stage till full implementation was found to be crucial for organizational change and enhancing ownership.

Moving from pilot to full-scale implementation requires intense groundwork for testing of the intervention holistically and is an iterative process which has been highlighted in this initiative. Lessons from the pilot/scalable unit provide the foundation for full scale-up of the intervention in a variety of settings and context. However, this study does not undermine the importance of going to full-scale experiential learning as the number of sites replicating interventions increase and progress from conception to full-scale.

During the exploration, installation and initial implementation stages identification of potential champions and best practices for the intervention supported effective delivery of Implementation Team’s work.

Selection of useable innovation by the Implementation Team based on its merit, simplicity, acceptability by the practitioners was another significant learning from this initiative. Active and timely communication between leadership and implementers was found to be crucial to understanding the difference between simple awareness raising of a new practice/innovation and what it takes to lead and ensure its adoption in full spirit with explicitly documented implementation policies. Furthermore, fostering an environment in which data-sharing in and within departments is the norm needs to be encouraged, such that beneficial outcomes can be measured.

Implementation science and practice are critical to the effective uptake of useable innovations and achieving program outcomes. This identifies a clear need to include implementation competencies in the training of clinicians and public health professionals working in MNCH. Once a mass of trained public health professionals i.e. policy makers and practitioners are created improvement in health indicators will be achievable. HSA can be trail blazer for developing such trainings and providing support to public health professionals at sub-national, regional and local levels.

**Box 1.** Defining SCC according to the Useable Innovation [4] criteria.

1. **Description of the Innovation**	1. **Essential Functions that Define the Innovation**
*Philosophy, values, and principles* Humane Effective Adoptable, Adaptable, Affordable Guiding Quality focused Replicable Individualized Inclusion and Exclusion Criteria Pregnant women of all ages, class and ethnicity without any mental or physical disorder visiting hospital to give birth will be included All booked and non-booked pregnant women without any mental or physical disorder visiting hospital to give birth will be included	The WHO Safe Childbirth Checklist is intended for use at four pause points during facility-based births: PAUSE POINT 1: ON ADMISSION PAUSE POINT 2: JUST BEFORE PUSHING (or before Caesarean) PAUSE POINT 3: SOON AFTER BIRTH (within one hour) PAUSE POINT 4: BEFORE DISCHARGE
2. **Core Components of the innovation and operation definitions**	
Universal: Essential birth practices are performed at critical moments during childbirth **for every delivery, every time** Practical: Checklist list comprises of a **core set of practices** that have been proven to reduce harm to mothers and newborns Remembrance: To prompt users to **remember essential tasks** to deliver better and safer care. SCC reminds the health care providers to follow certain procedure during the delivery process (not to forget important essential steps) Ensuing or following: If the pause points take place in separate locations, then the Checklist must **‘follow’** the mother and newborn as they move from room to room User friendly: Checklists can commonly be used in two ways: in ‘Read-Do,’ first read the item on the Checklist, then complete the task. In ‘Do-Confirm,’ complete the task then read the item on the Checklist to confirm that you have done it	

## Paper context

Implementation science and practice as part of professional training is yet to be realized in Pakistan. To fill this gap, an online course was attended by individuals working in public health. Aim was to develop implementation science capacity. The 52-week course with online teaching, coaching, and a project, was completed by 8 individuals. The outcome is an Implementation Team trained in use of Active Implementation Frameworks.
